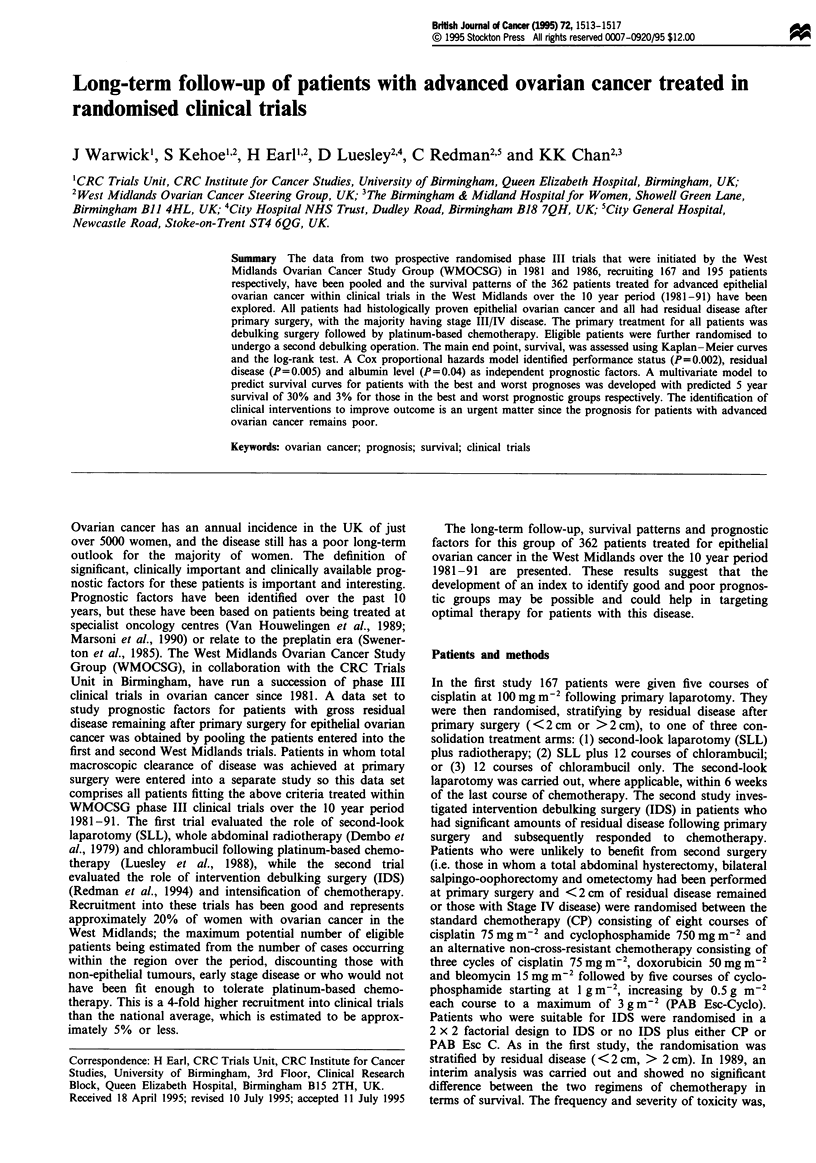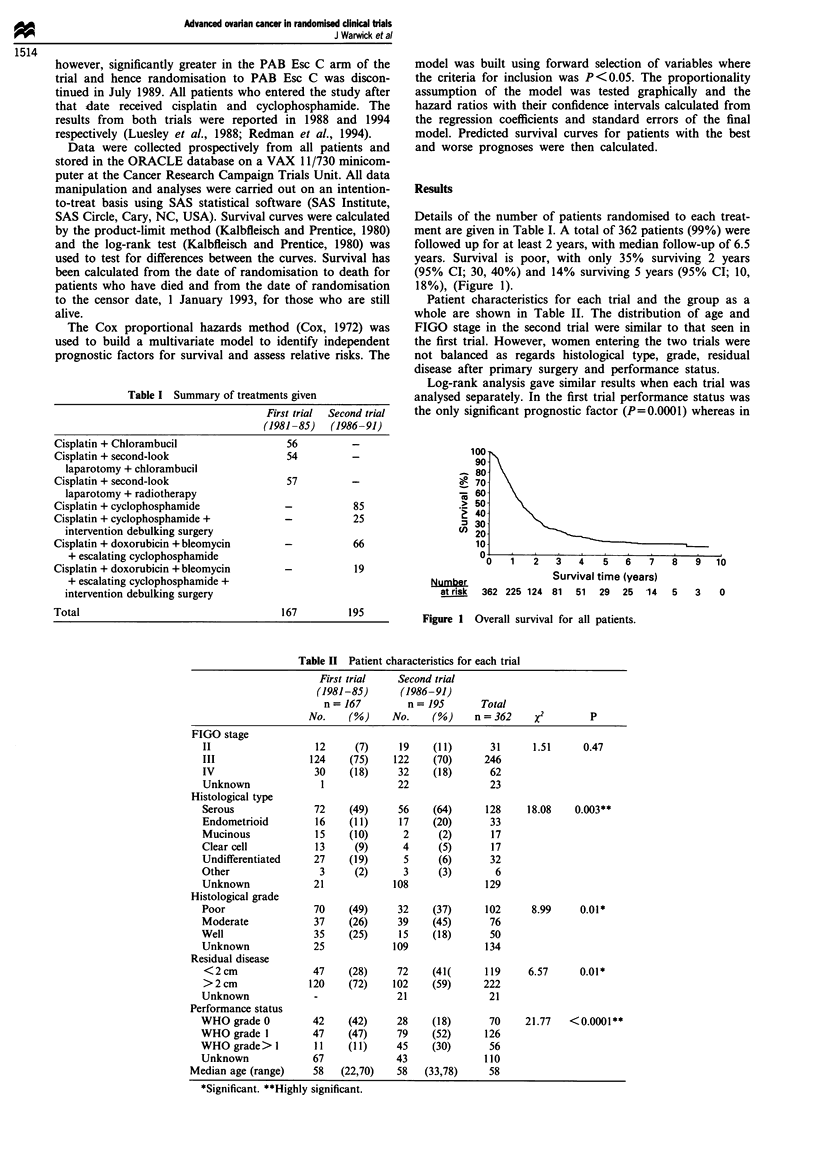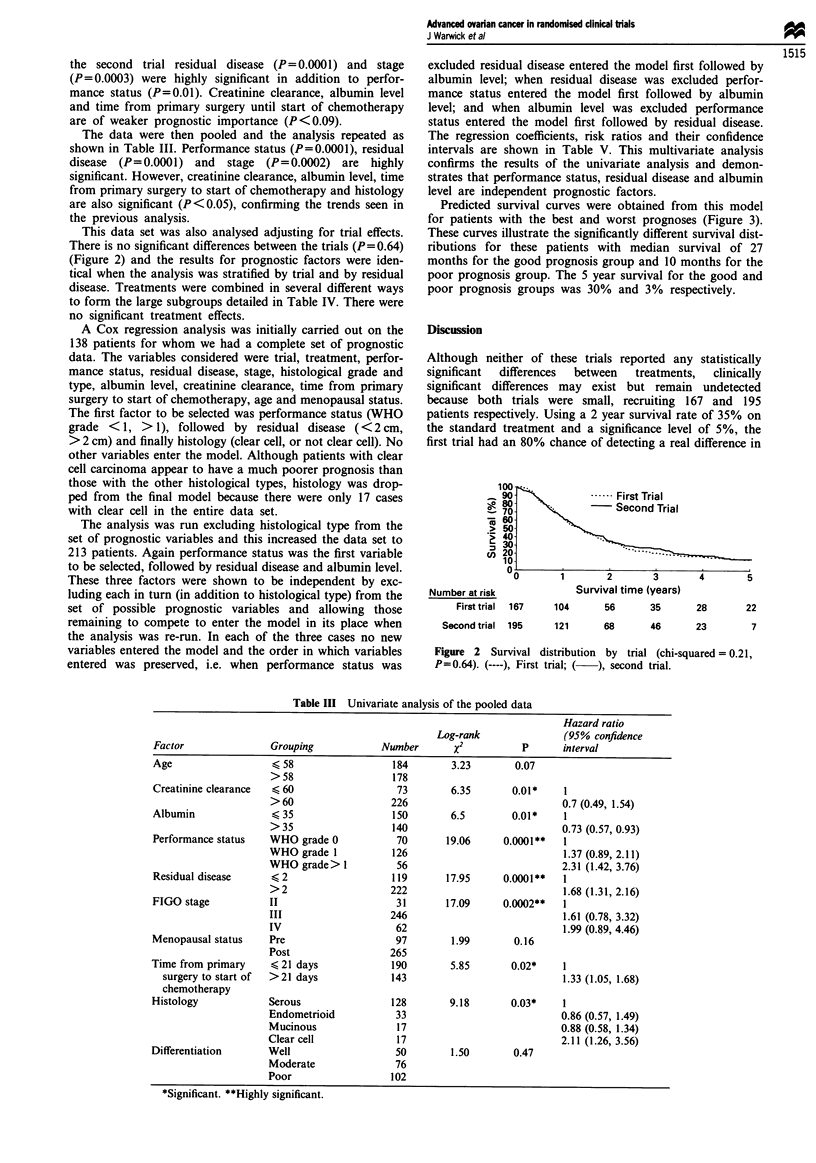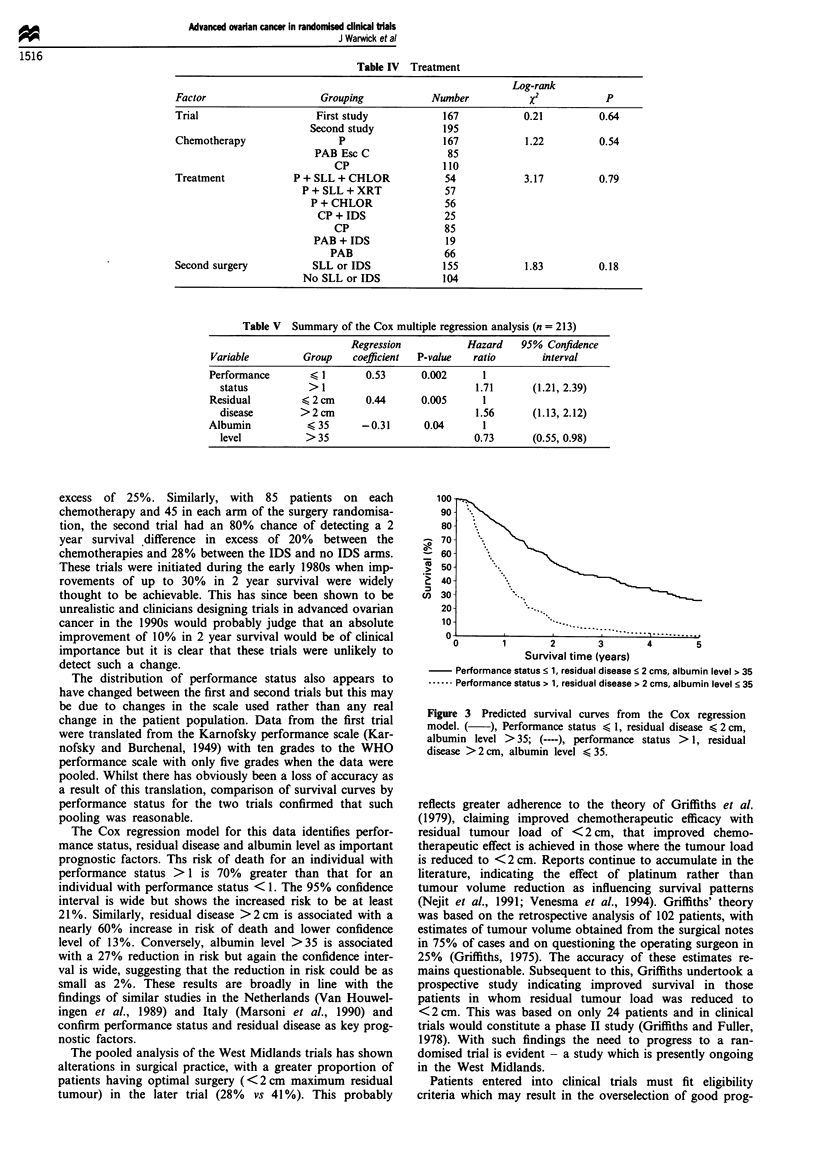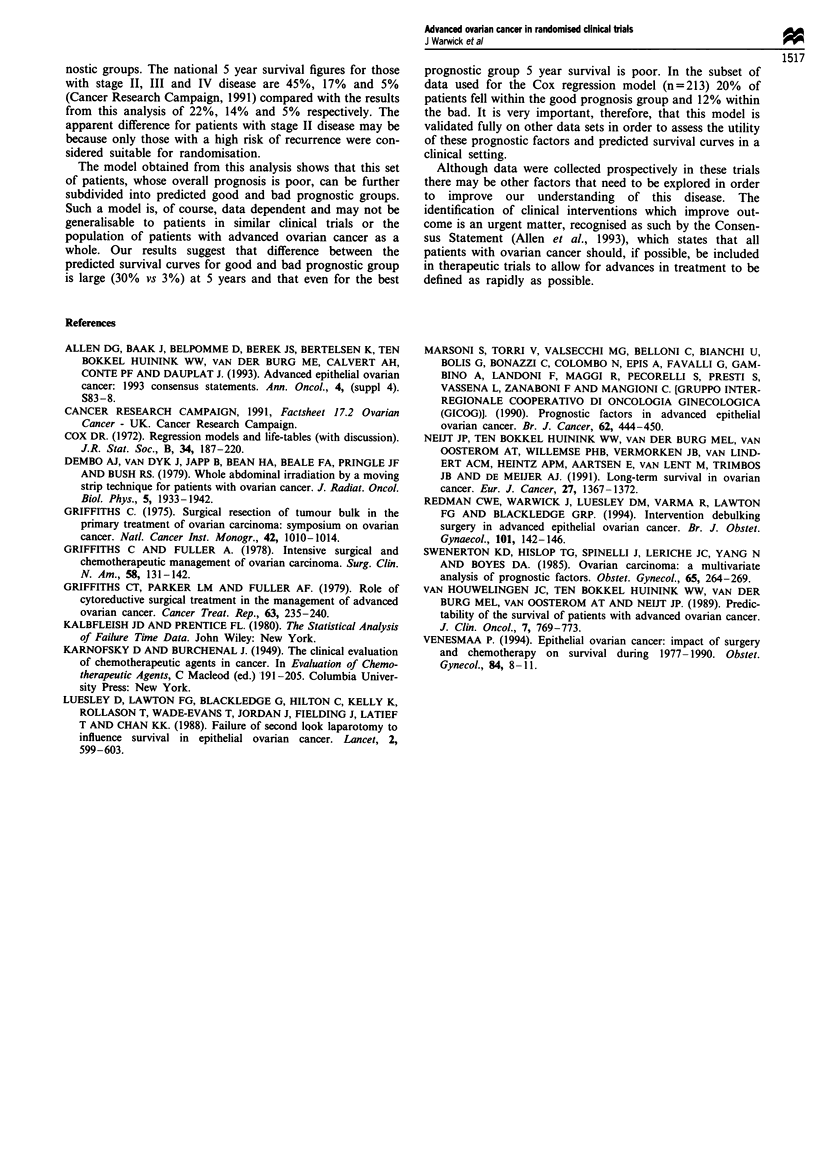# Long-term follow-up of patients with advanced ovarian cancer treated in randomised clinical trials.

**DOI:** 10.1038/bjc.1995.539

**Published:** 1995-12

**Authors:** J. Warwick, S. Kehoe, H. Earl, D. Luesley, C. Redman, K. K. Chan

**Affiliations:** CRC Trials Unit, CRC Institute for Cancer Studies, University of Birmingham, Queen Elizabeth Hospital, UK.

## Abstract

The data from two prospective randomised phase III trials that were initiated by the West Midlands Ovarian Cancer Study Group (WMOCSG) in 1981 and 1986, recruiting 167 and 195 patients respectively, have been pooled and the survival patterns of the 362 patients treated for advanced epithelial ovarian cancer within clinical trials in the West Midlands over the 10 year period (1981-91) have been explored. All patients had histologically proven epithelial ovarian cancer and all had residual disease after primary surgery, with the majority having stage III/IV disease. The primary treatment for all patients was debulking surgery followed by platinum-based chemotherapy. Eligible patients were further randomised to undergo a second debulking operation. The main end point, survival, was assessed using Kaplan-Meier curves and the log-rank test. A Cox proportional hazards model identified performance status (P = 0.002), residual disease (P = 0.005) and albumin level (P = 0.04) as independent prognostic factors. A multivariate model to predict survival curves for patients with the best and worst prognoses was developed with predicted 5 year survival of 30% and 3% for those in the best and worst prognostic groups respectively. The identification of clinical interventions to improve outcome is an urgent matter since the prognosis for patients with advanced ovarian cancer remains poor.


British Journal of Cancer (1995) 72, 1513-1517
© 1995 Stockton Press All rights reserved 0007-0920/95 $12.00

### Long-term follow-up of patients with advanced ovarian cancer treated in randomised clinical trials

J Warwick[1], S Kehoe[1,2], H Earl[1,2], D Luesley[2,4], C Redman[2,5] and KK Chan[2,3]

[1]*CRC Trials Unit, CRC Institute for Cancer Studies, University of Birmingham, Queen Elizabeth Hospital, Birmingham, UK;* [2]*West Midlands Ovarian Cancer Steering Group, UK;* [3]*The Birmingham & Midland Hospital for Women, Showell Green Lane, Birmingham B11 4HL, UK;* [4]*City Hospital NHS Trust, Dudley Road, Birmingham B18 7QH, UK;* [5]*City General Hospital, Newcastle Road, Stoke-on-Trent ST4 6QG, UK.*

**Summary** The data from two prospective randomised phase III trials that were initiated by the West Midlands Ovarian Cancer Study Group (WMOCSG) in 1981 and 1986, recruiting 167 and 195 patients respectively, have been pooled and the survival patterns of the 362 patients treated for advanced epithelial ovarian cancer within clinical trials in the West Midlands over the 10 year period (1981-91) have been explored. All patients had histologically proven epithelial ovarian cancer and all had residual disease after primary surgery, with the majority having stage III/IV disease. The primary treatment for all patients was debulking surgery followed by platinum-based chemotherapy. Eligible patients were further randomised to undergo a second debulking operation. The main end point, survival, was assessed using Kaplan-Meier curves and the log-rank test. A Cox proportional hazards model identified performance status ($P = 0.002$), residual disease ($P = 0.005$) and albumin level ($P = 0.04$) as independent prognostic factors. A multivariate model to predict survival curves for patients with the best and worst prognoses was developed with predicted 5 year survival of 30% and 3% for those in the best and worst prognostic groups respectively. The identification of clinical interventions to improve outcome is an urgent matter since the prognosis for patients with advanced ovarian cancer remains poor.

**Keywords:** ovarian cancer; prognosis; survival; clinical trials

Ovarian cancer has an annual incidence in the UK of just over 5000 women, and the disease still has a poor long-term outlook for the majority of women. The definition of significant, clinically important and clinically available prognostic factors for these patients is important and interesting. Prognostic factors have been identified over the past 10 years, but these have been based on patients being treated at specialist oncology centres (Van Houwelingen *et al.*, 1989; Marsoni *et al.*, 1990) or relate to the preplatin era (Swenerton *et al.*, 1985). The West Midlands Ovarian Cancer Study Group (WMOCSG), in collaboration with the CRC Trials Unit in Birmingham, have run a succession of phase III clinical trials in ovarian cancer since 1981. A data set to study prognostic factors for patients with gross residual disease remaining after primary surgery for epithelial ovarian cancer was obtained by pooling the patients entered into the first and second West Midlands trials. Patients in whom total macroscopic clearance of disease was achieved at primary surgery were entered into a separate study so this data set comprises all patients fitting the above criteria treated within WMOCSG phase III clinical trials over the 10 year period 1981-91. The first trial evaluated the role of second-look laparotomy (SLL), whole abdominal radiotherapy (Dembo *et al.*, 1979) and chlorambucil following platinum-based chemotherapy (Luesley *et al.*, 1988), while the second trial evaluated the role of intervention debulking surgery (IDS) (Redman *et al.*, 1994) and intensification of chemotherapy. Recruitment into these trials has been good and represents approximately 20% of women with ovarian cancer in the West Midlands; the maximum potential number of eligible patients being estimated from the number of cases occurring within the region over the period, discounting those with non-epithelial tumours, early stage disease or who would not have been fit enough to tolerate platinum-based chemotherapy. This is a 4-fold higher recruitment into clinical trials than the national average, which is estimated to be approximately 5% or less.

Correspondence: H Earl, CRC Trials Unit, CRC Institute for Cancer Studies, University of Birmingham, 3rd Floor, Clinical Research Block, Queen Elizabeth Hospital, Birmingham B15 2TH, UK.
Received 18 April 1995; revised 10 July 1995; accepted 11 July 1995

The long-term follow-up, survival patterns and prognostic factors for this group of 362 patients treated for epithelial ovarian cancer in the West Midlands over the 10 year period 1981-91 are presented. These results suggest that the development of an index to identify good and poor prognostic groups may be possible and could help in targeting optimal therapy for patients with this disease.

### Patients and methods

In the first study 167 patients were given five courses of cisplatin at 100 mg m$^{-2}$ following primary laparotomy. They were then randomised, stratifying by residual disease after primary surgery (<2 cm or >2 cm), to one of three consolidation treatment arms: (1) second-look laparotomy (SLL) plus radiotherapy; (2) SLL plus 12 courses of chlorambucil; or (3) 12 courses of chlorambucil only. The second-look laparotomy was carried out, where applicable, within 6 weeks of the last course of chemotherapy. The second study investigated intervention debulking surgery (IDS) in patients who had significant amounts of residual disease following primary surgery and subsequently responded to chemotherapy. Patients who were unlikely to benefit from second surgery (i.e. those in whom a total abdominal hysterectomy, bilateral salpingo-oophorectomy and ometectomy had been performed at primary surgery and <2 cm of residual disease remained or those with Stage IV disease) were randomised between the standard chemotherapy (CP) consisting of eight courses of cisplatin 75 mg m$^{-2}$ and cyclophosphamide 750 mg m$^{-2}$ and an alternative non-cross-resistant chemotherapy consisting of three cycles of cisplatin 75 mg m$^{-2}$, doxorubicin 50 mg m$^{-2}$ and bleomycin 15 mg m$^{-2}$ followed by five courses of cyclophosphamide starting at 1 g m$^{-2}$, increasing by 0.5 g m$^{-2}$ each course to a maximum of 3 g m$^{-2}$ (PAB Esc-Cyclo). Patients who were suitable for IDS were randomised in a 2 x 2 factorial design to IDS or no IDS plus either CP or PAB Esc C. As in the first study, the randomisation was stratified by residual disease (<2 cm, > 2 cm). In 1989, an interim analysis was carried out and showed no significant difference between the two regimens of chemotherapy in terms of survival. The frequency and severity of toxicity was, however, significantly greater in the PAB Esc C arm of the trial and hence randomisation to PAB Esc C was discontinued in July 1989. All patients who entered the study after that date received cisplatin and cyclophosphamide. The results from both trials were reported in 1988 and 1994 respectively (Luesley *et al.*, 1988; Redman *et al.*, 1994).

Data were collected prospectively from all patients and stored in the ORACLE database on a VAX 11/730 minicomputer at the Cancer Research Campaign Trials Unit. All data manipulation and analyses were carried out on an intention-to-treat basis using SAS statistical software (SAS Institute, SAS Circle, Cary, NC, USA). Survival curves were calculated by the product-limit method (Kalbfleisch and Prentice, 1980) and the log-rank test (Kalbfleisch and Prentice, 1980) was used to test for differences between the curves. Survival has been calculated from the date of randomisation to death for patients who have died and from the date of randomisation to the censor date, 1 January 1993, for those who are still alive.

The Cox proportional hazards method (Cox, 1972) was used to build a multivariate model to identify independent prognostic factors for survival and assess relative risks. The model was built using forward selection of variables where the criteria for inclusion was $P<0.05$. The proportionality assumption of the model was tested graphically and the hazard ratios with their confidence intervals calculated from the regression coefficients and standard errors of the final model. Predicted survival curves for patients with the best and worse prognoses were then calculated.

**Table I** Summary of treatments given

| | *First trial (1981–85)* | *Second trial (1986–91)* |
|---|---|---|
| Cisplatin + Chlorambucil | 56 | – |
| Cisplatin + second-look laparotomy + chlorambucil | 54 | – |
| Cisplatin + second-look laparotomy + radiotherapy | 57 | – |
| Cisplatin + cyclophosphamide | – | 85 |
| Cisplatin + cyclophosphamide + intervention debulking surgery | – | 25 |
| Cisplatin + doxorubicin + bleomycin + escalating cyclophosphamide | – | 66 |
| Cisplatin + doxorubicin + bleomycin + escalating cyclophosphamide + intervention debulking surgery | – | 19 |
| Total | 167 | 195 |

### Results

Details of the number of patients randomised to each treatment are given in Table I. A total of 362 patients (99%) were followed up for at least 2 years, with median follow-up of 6.5 years. Survival is poor, with only 35% surviving 2 years (95% CI; 30, 40%) and 14% surviving 5 years (95% CI; 10, 18%), (Figure 1).

Patient characteristics for each trial and the group as a whole are shown in Table II. The distribution of age and FIGO stage in the second trial were similar to that seen in the first trial. However, women entering the two trials were not balanced as regards histological type, grade, residual disease after primary surgery and performance status.

Log-rank analysis gave similar results when each trial was analysed separately. In the first trial performance status was the only significant prognostic factor ($P=0.0001$) whereas in

**Figure 1** Overall survival for all patients.

**Table II** Patient characteristics for each trial

| | *First trial (1981–85)* n = *167* *No.* | *(%)* | *Second trial (1986–91)* n = *195* *No.* | *(%)* | *Total* n = *362* | $\chi^2$ | P |
|---|---|---|---|---|---|---|---|
| FIGO stage | | | | | | | |
| II | 12 | (7) | 19 | (11) | 31 | 1.51 | 0.47 |
| III | 124 | (75) | 122 | (70) | 246 | | |
| IV | 30 | (18) | 32 | (18) | 62 | | |
| Unknown | 1 | | 22 | | 23 | | |
| Histological type | | | | | | | |
| Serous | 72 | (49) | 56 | (64) | 128 | 18.08 | 0.003** |
| Endometrioid | 16 | (11) | 17 | (20) | 33 | | |
| Mucinous | 15 | (10) | 2 | (2) | 17 | | |
| Clear cell | 13 | (9) | 4 | (5) | 17 | | |
| Undifferentiated | 27 | (19) | 5 | (6) | 32 | | |
| Other | 3 | (2) | 3 | (3) | 6 | | |
| Unknown | 21 | | 108 | | 129 | | |
| Histological grade | | | | | | | |
| Poor | 70 | (49) | 32 | (37) | 102 | 8.99 | 0.01* |
| Moderate | 37 | (26) | 39 | (45) | 76 | | |
| Well | 35 | (25) | 15 | (18) | 50 | | |
| Unknown | 25 | | 109 | | 134 | | |
| Residual disease | | | | | | | |
| <2 cm | 47 | (28) | 72 | (41( | 119 | 6.57 | 0.01* |
| >2 cm | 120 | (72) | 102 | (59) | 222 | | |
| Unknown | - | | 21 | | 21 | | |
| Performance status | | | | | | | |
| WHO grade 0 | 42 | (42) | 28 | (18) | 70 | 21.77 | <0.0001** |
| WHO grade 1 | 47 | (47) | 79 | (52) | 126 | | |
| WHO grade > 1 | 11 | (11) | 45 | (30) | 56 | | |
| Unknown | 67 | | 43 | | 110 | | |
| Median age (range) | 58 | (22,70) | 58 | (33,78) | 58 | | |

*Significant. **Highly significant.

the second trial residual disease ($P=0.0001$) and stage ($P=0.0003$) were highly significant in addition to performance status ($P=0.01$). Creatinine clearance, albumin level and time from primary surgery until start of chemotherapy are of weaker prognostic importance ($P<0.09$).

The data were then pooled and the analysis repeated as shown in Table III. Performance status ($P=0.0001$), residual disease ($P=0.0001$) and stage ($P=0.0002$) are highly significant. However, creatinine clearance, albumin level, time from primary surgery to start of chemotherapy and histology are also significant ($P<0.05$), confirming the trends seen in the previous analysis.

This data set was also analysed adjusting for trial effects. There is no significant differences between the trials ($P=0.64$) (Figure 2) and the results for prognostic factors were identical when the analysis was stratified by trial and by residual disease. Treatments were combined in several different ways to form the large subgroups detailed in Table IV. There were no significant treatment effects.

A Cox regression analysis was initially carried out on the 138 patients for whom we had a complete set of prognostic data. The variables considered were trial, treatment, performance status, residual disease, stage, histological grade and type, albumin level, creatinine clearance, time from primary surgery to start of chemotherapy, age and menopausal status. The first factor to be selected was performance status (WHO grade $<1$, $>1$), followed by residual disease ($<2$ cm, $>2$ cm) and finally histology (clear cell, or not clear cell). No other variables enter the model. Although patients with clear cell carcinoma appear to have a much poorer prognosis than those with the other histological types, histology was dropped from the final model because there were only 17 cases with clear cell in the entire data set.

The analysis was run excluding histological type from the set of prognostic variables and this increased the data set to 213 patients. Again performance status was the first variable to be selected, followed by residual disease and albumin level. These three factors were shown to be independent by excluding each in turn (in addition to histological type) from the set of possible prognostic variables and allowing those remaining to compete to enter the model in its place when the analysis was re-run. In each of the three cases no new variables entered the model and the order in which variables entered was preserved, i.e. when performance status was excluded residual disease entered the model first followed by albumin level; when residual disease was excluded performance status entered the model first followed by albumin level; and when albumin level was excluded performance status entered the model first followed by residual disease. The regression coefficients, risk ratios and their confidence intervals are shown in Table V. This multivariate analysis confirms the results of the univariate analysis and demonstrates that performance status, residual disease and albumin level are independent prognostic factors.

Predicted survival curves were obtained from this model for patients with the best and worst prognoses (Figure 3). These curves illustrate the significantly different survival distributions for these patients with median survival of 27 months for the good prognosis group and 10 months for the poor prognosis group. The 5 year survival for the good and poor prognosis groups was 30% and 3% respectively.

### Discussion

Although neither of these trials reported any statistically significant differences between treatments, clinically significant differences may exist but remain undetected because both trials were small, recruiting 167 and 195 patients respectively. Using a 2 year survival rate of 35% on the standard treatment and a significance level of 5%, the first trial had an 80% chance of detecting a real difference in

**Figure 2** Survival distribution by trial (chi-squared = 0.21, $P=0.64$). (----), First trial; (——), second trial.

**Table III** Univariate analysis of the pooled data

| *Factor* | *Grouping* | *Number* | *Log-rank* $\chi^2$ | P | *Hazard ratio (95% confidence interval* |
|---|---|---|---|---|---|
| Age | ≤58 | 184 | 3.23 | 0.07 | |
| | >58 | 178 | | | |
| Creatinine clearance | ≤60 | 73 | 6.35 | 0.01* | 1 |
| | >60 | 226 | | | 0.7 (0.49, 1.54) |
| Albumin | ≤35 | 150 | 6.5 | 0.01* | 1 |
| | >35 | 140 | | | 0.73 (0.57, 0.93) |
| Performance status | WHO grade 0 | 70 | 19.06 | 0.0001** | 1 |
| | WHO grade 1 | 126 | | | 1.37 (0.89, 2.11) |
| | WHO grade >1 | 56 | | | 2.31 (1.42, 3.76) |
| Residual disease | ≤2 | 119 | 17.95 | 0.0001** | 1 |
| | >2 | 222 | | | 1.68 (1.31, 2.16) |
| FIGO stage | II | 31 | 17.09 | 0.0002** | 1 |
| | III | 246 | | | 1.61 (0.78, 3.32) |
| | IV | 62 | | | 1.99 (0.89, 4.46) |
| Menopausal status | Pre | 97 | 1.99 | 0.16 | |
| | Post | 265 | | | |
| Time from primary surgery to start of chemotherapy | ≤21 days | 190 | 5.85 | 0.02* | 1 |
| | >21 days | 143 | | | 1.33 (1.05, 1.68) |
| Histology | Serous | 128 | 9.18 | 0.03* | 1 |
| | Endometrioid | 33 | | | 0.86 (0.57, 1.49) |
| | Mucinous | 17 | | | 0.88 (0.58, 1.34) |
| | Clear cell | 17 | | | 2.11 (1.26, 3.56) |
| Differentiation | Well | 50 | 1.50 | 0.47 | |
| | Moderate | 76 | | | |
| | Poor | 102 | | | |

*Significant. **Highly significant.

**Table IV** Treatment

| Factor | Grouping | Number | Log-rank $\chi^2$ | P |
|---|---|---|---|---|
| Trial | First study | 167 | 0.21 | 0.64 |
| | Second study | 195 | | |
| Chemotherapy | P | 167 | 1.22 | 0.54 |
| | PAB Esc C | 85 | | |
| | CP | 110 | | |
| Treatment | P + SLL + CHLOR | 54 | 3.17 | 0.79 |
| | P + SLL + XRT | 57 | | |
| | P + CHLOR | 56 | | |
| | CP + IDS | 25 | | |
| | CP | 85 | | |
| | PAB + IDS | 19 | | |
| | PAB | 66 | | |
| Second surgery | SLL or IDS | 155 | 1.83 | 0.18 |
| | No SLL or IDS | 104 | | |

**Table V** Summary of the Cox multiple regression analysis ($n = 213$)

| Variable | Group | Regression coefficient | P-value | Hazard ratio | 95% Confidence interval |
|---|---|---|---|---|---|
| Performance | ⩽1 | 0.53 | 0.002 | 1 | |
| status | >1 | | | 1.71 | (1.21, 2.39) |
| Residual | ⩽2 cm | 0.44 | 0.005 | 1 | |
| disease | >2 cm | | | 1.56 | (1.13, 2.12) |
| Albumin | ⩽35 | −0.31 | 0.04 | 1 | |
| level | >35 | | | 0.73 | (0.55, 0.98) |

excess of 25%. Similarly, with 85 patients on each chemotherapy and 45 in each arm of the surgery randomisation, the second trial had an 80% chance of detecting a 2 year survival difference in excess of 20% between the chemotherapies and 28% between the IDS and no IDS arms. These trials were initiated during the early 1980s when improvements of up to 30% in 2 year survival were widely thought to be achievable. This has since been shown to be unrealistic and clinicians designing trials in advanced ovarian cancer in the 1990s would probably judge that an absolute improvement of 10% in 2 year survival would be of clinical importance but it is clear that these trials were unlikely to detect such a change.

The distribution of performance status also appears to have changed between the first and second trials but this may be due to changes in the scale used rather than any real change in the patient population. Data from the first trial were translated from the Karnofsky performance scale (Karnofsky and Burchenal, 1949) with ten grades to the WHO performance scale with only five grades when the data were pooled. Whilst there has obviously been a loss of accuracy as a result of this translation, comparison of survival curves by performance status for the two trials confirmed that such pooling was reasonable.

The Cox regression model for this data identifies performance status, residual disease and albumin level as important prognostic factors. Ths risk of death for an individual with performance status >1 is 70% greater than that for an individual with performance status <1. The 95% confidence interval is wide but shows the increased risk to be at least 21%. Similarly, residual disease >2 cm is associated with a nearly 60% increase in risk of death and lower confidence level of 13%. Conversely, albumin level >35 is associated with a 27% reduction in risk but again the confidence interval is wide, suggesting that the reduction in risk could be as small as 2%. These results are broadly in line with the findings of similar studies in the Netherlands (Van Houwelingen *et al.*, 1989) and Italy (Marsoni *et al.*, 1990) and confirm performance status and residual disease as key prognostic factors.

The pooled analysis of the West Midlands trials has shown alterations in surgical practice, with a greater proportion of patients having optimal surgery (<2 cm maximum residual tumour) in the later trial (28% *vs* 41%). This probably reflects greater adherence to the theory of Griffiths *et al.* (1979), claiming improved chemotherapeutic efficacy with residual tumour load of <2 cm, that improved chemotherapeutic effect is achieved in those where the tumour load is reduced to <2 cm. Reports continue to accumulate in the literature, indicating the effect of platinum rather than tumour volume reduction as influencing survival patterns (Nejit *et al.*, 1991; Venesma *et al.*, 1994). Griffiths' theory was based on the retrospective analysis of 102 patients, with estimates of tumour volume obtained from the surgical notes in 75% of cases and on questioning the operating surgeon in 25% (Griffiths, 1975). The accuracy of these estimates remains questionable. Subsequent to this, Griffiths undertook a prospective study indicating improved survival in those patients in whom residual tumour load was reduced to <2 cm. This was based on only 24 patients and in clinical trials would constitute a phase II study (Griffiths and Fuller, 1978). With such findings the need to progress to a randomised trial is evident – a study which is presently ongoing in the West Midlands.

**Figure 3** Predicted survival curves from the Cox regression model. (——), Performance status ⩽1, residual disease ⩽2 cm, albumin level >35; (----), performance status >1, residual disease >2 cm, albumin level ⩽35.

Patients entered into clinical trials must fit eligibility criteria which may result in the overselection of good prognostic groups. The national 5 year survival figures for those with stage II, III and IV disease are 45%, 17% and 5% (Cancer Research Campaign, 1991) compared with the results from this analysis of 22%, 14% and 5% respectively. The apparent difference for patients with stage II disease may be because only those with a high risk of recurrence were considered suitable for randomisation.

The model obtained from this analysis shows that this set of patients, whose overall prognosis is poor, can be further subdivided into predicted good and bad prognostic groups. Such a model is, of course, data dependent and may not be generalisable to patients in similar clinical trials or the population of patients with advanced ovarian cancer as a whole. Our results suggest that difference between the predicted survival curves for good and bad prognostic group is large (30% *vs* 3%) at 5 years and that even for the best prognostic group 5 year survival is poor. In the subset of data used for the Cox regression model (n=213) 20% of patients fell within the good prognosis group and 12% within the bad. It is very important, therefore, that this model is validated fully on other data sets in order to assess the utility of these prognostic factors and predicted survival curves in a clinical setting.

Although data were collected prospectively in these trials there may be other factors that need to be explored in order to improve our understanding of this disease. The identification of clinical interventions which improve outcome is an urgent matter, recognised as such by the Consensus Statement (Allen *et al.*, 1993), which states that all patients with ovarian cancer should, if possible, be included in therapeutic trials to allow for advances in treatment to be defined as rapidly as possible.

### References

ALLEN DG, BAAK J, BELPOMME D, BEREK JS, BERTELSEN K, TEN BOKKEL HUININK WW, VAN DER BURG ME, CALVERT AH, CONTE PF AND DAUPLAT J. (1993). Advanced epithelial ovarian cancer: 1993 consensus statements. *Ann. Oncol.*, **4**, (suppl 4). S83-8.

CANCER RESEARCH CAMPAIGN, 1991, *Factsheet 17.2 Ovarian Cancer* - UK. Cancer Research Campaign.

COX DR. (1972). Regression models and life-tables (with discussion). *J.R. Stat. Soc.*, B, **34**, 187-220.

DEMBO AJ, VAN DYK J, JAPP B, BEAN HA, BEALE FA, PRINGLE JF AND BUSH RS. (1979). Whole abdominal irradiation by a moving strip technique for patients with ovarian cancer. *J. Radiat. Oncol. Biol. Phys.*, **5**, 1933-1942.

GRIFFITHS C. (1975). Surgical resection of tumour bulk in the primary treatment of ovarian carcinoma: symposium on ovarian cancer. *Natl. Cancer Inst. Monogr.*, **42**, 1010-1014.

GRIFFITHS C AND FULLER A. (1978). Intensive surgical and chemotherapeutic management of ovarian carcinoma. *Surg. Clin. N. Am.*, **58**, 131-142.

GRIFFITHS CT, PARKER LM AND FULLER AF. (1979). Role of cytoreductive surgical treatment in the management of advanced ovarian cancer. *Cancer Treat. Rep.*, **63**, 235-240.

KALBFLEISH JD AND PRENTICE FL. (1980). *The Statistical Analysis of Failure Time Data.* John Wiley: New York.

KARNOFSKY D AND BURCHENAL J. (1949). The clinical evaluation of chemotherapeutic agents in cancer. In *Evaluation of Chemotherapeutic Agents*, C Macleod (ed.) 191-205. Columbia University Press: New York.

LUESLEY D, LAWTON FG, BLACKLEDGE G, HILTON C, KELLY K, ROLLASON T, WADE-EVANS T, JORDAN J, FIELDING J, LATIEF T AND CHAN KK. (1988). Failure of second look laparotomy to influence survival in epithelial ovarian cancer. *Lancet*, **2**, 599-603.

MARSONI S, TORRI V, VALSECCHI MG, BELLONI C, BIANCHI U, BOLIS G, BONAZZI C, COLOMBO N, EPIS A, FAVALLI G, GAMBINO A, LANDONI F, MAGGI R, PECORELLI S, PRESTI S, VASSENA L, ZANABONI F AND MANGIONI C. [GRUPPO INTERREGIONALE COOPERATIVO DI ONCOLOGIA GINECOLOGICA (GICOG)]. (1990). Prognostic factors in advanced epithelial ovarian cancer. *Br. J. Cancer*, **62**, 444-450.

NEIJT JP, TEN BOKKEL HUININK WW, VAN DER BURG MEL, VAN OOSTEROM AT, WILLEMSE PHB, VERMORKEN JB, VAN LINDERT ACM, HEINTZ APM, AARTSEN E, VAN LENT M, TRIMBOS JB AND DE MEIJER AJ. (1991). Long-term survival in ovarian cancer. *Eur. J. Cancer*, **27**, 1367-1372.

REDMAN CWE, WARWICK J, LUESLEY DM, VARMA R, LAWTON FG AND BLACKLEDGE GRP. (1994). Intervention debulking surgery in advanced epithelial ovarian cancer. *Br. J. Obstet. Gynaecol.*, **101**, 142-146.

SWENERTON KD, HISLOP TG, SPINELLI J, LERICHE JC, YANG N AND BOYES DA. (1985). Ovarian carcinoma: a multivariate analysis of prognostic factors. *Obstet. Gynecol.*, **65**, 264-269.

VAN HOUWELINGEN JC, TEN BOKKEL HUININK WW, VAN DER BURG MEL, VAN OOSTEROM AT AND NEIJT JP. (1989). Predictability of the survival of patients with advanced ovarian cancer. *J. Clin. Oncol.*, **7**, 769-773.

VENESMAA P. (1994). Epithelial ovarian cancer: impact of surgery and chemotherapy on survival during 1977-1990. *Obstet. Gynecol.*, **84**, 8-11.